# Supplementary figures and images for: Human Dectin-1 is O-glycosylated and serves as a ligand for C-type lectin receptor CLEC-2
Source: eLife. 2022 Dec 8;11:e83037. doi: 10.7554/eLife.83037 (PMC9788829; doi:10.7554/eLife.83037)

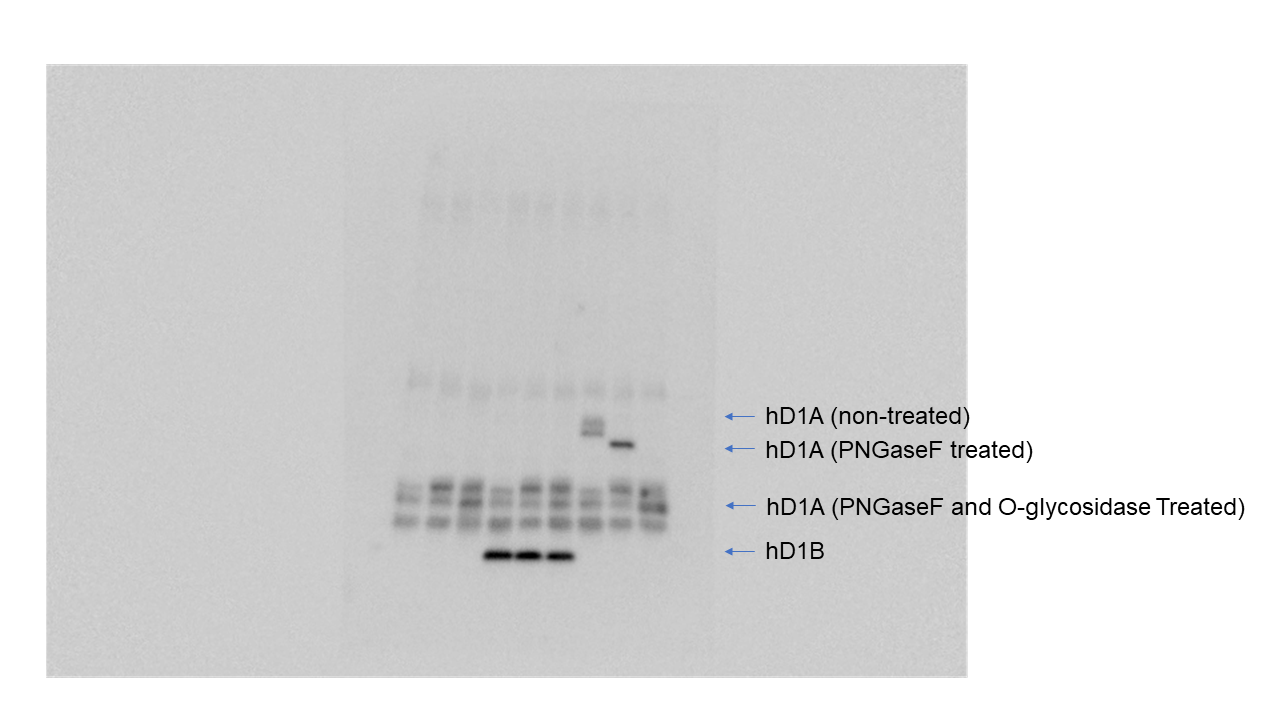

Supplement: Figure 4—source data 1. [file elife-83037-fig4-data1.zip › Figure 4-source data 1 indicated.tif]

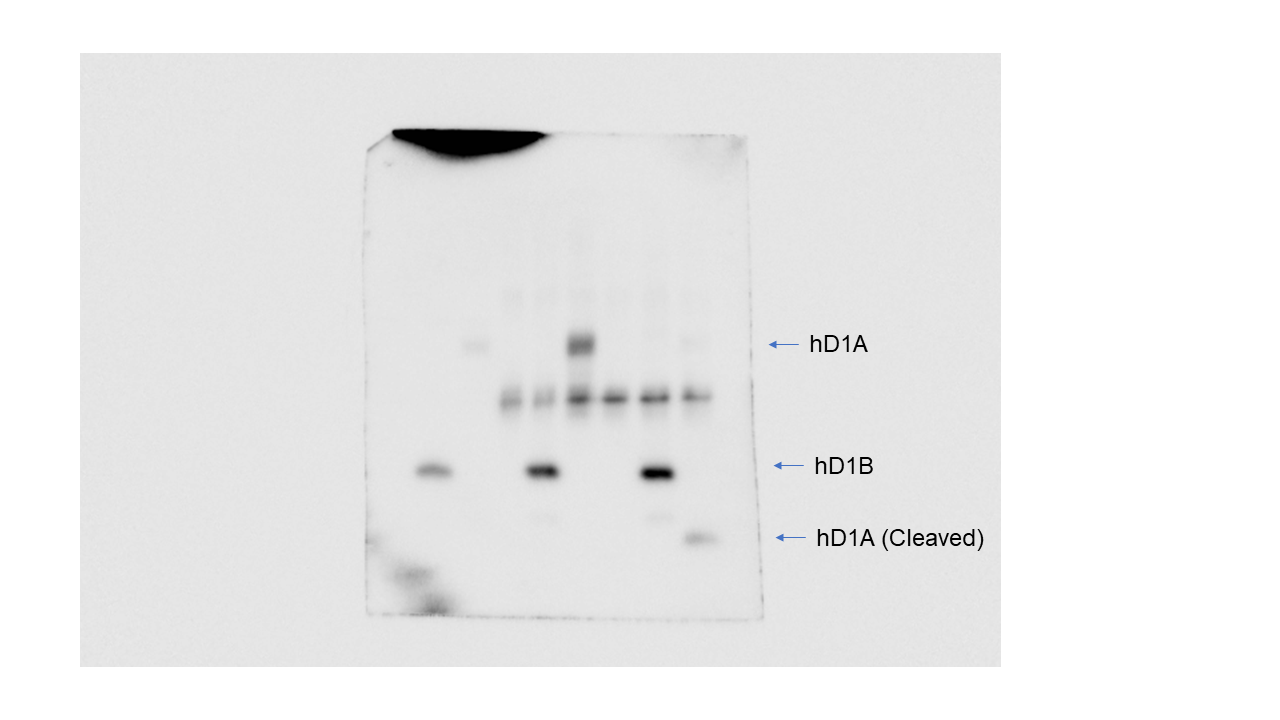

Supplement: Figure 4—source data 2. [file elife-83037-fig4-data2.zip › Figure 4-source data 2 indicated.tif]

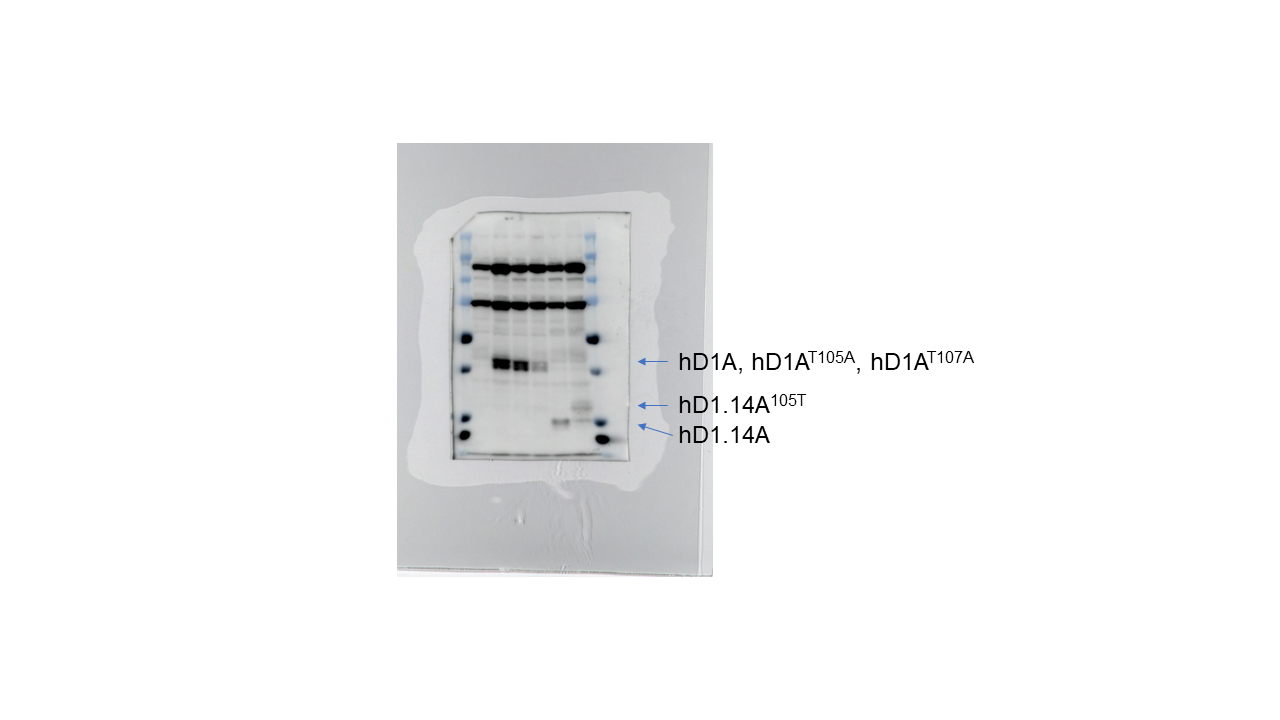

Supplement: Figure 5—figure supplement 1—source data 1. [file elife-83037-fig5-figsupp1-data1.zip › Figure 5-figure supplement 1-source data 1 1 indicated.TIF]

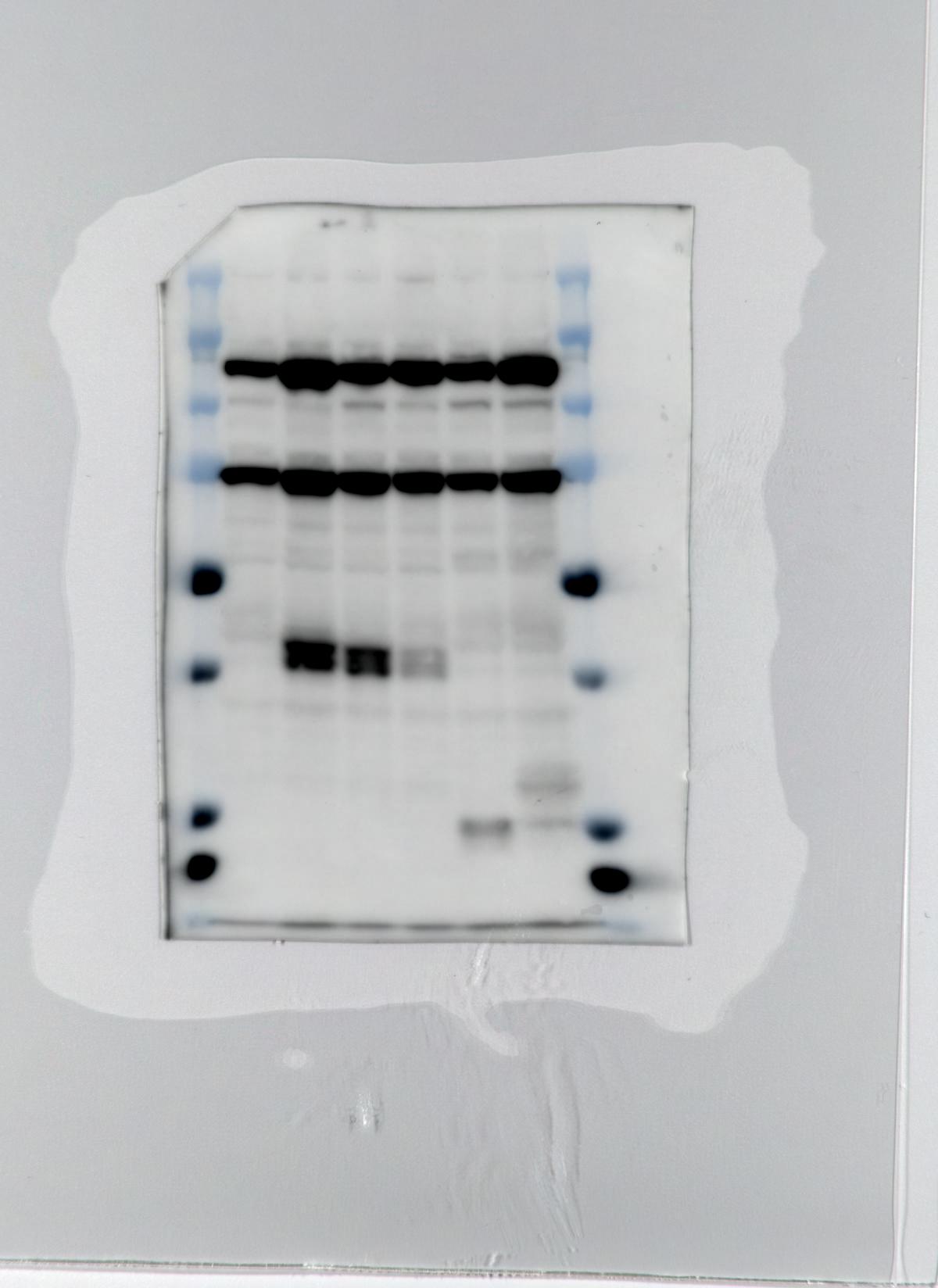

Supplement: Figure 5—figure supplement 1—source data 1. [file elife-83037-fig5-figsupp1-data1.zip › Figure 5-figure supplement 1-source data 1 1 raw.jpg]

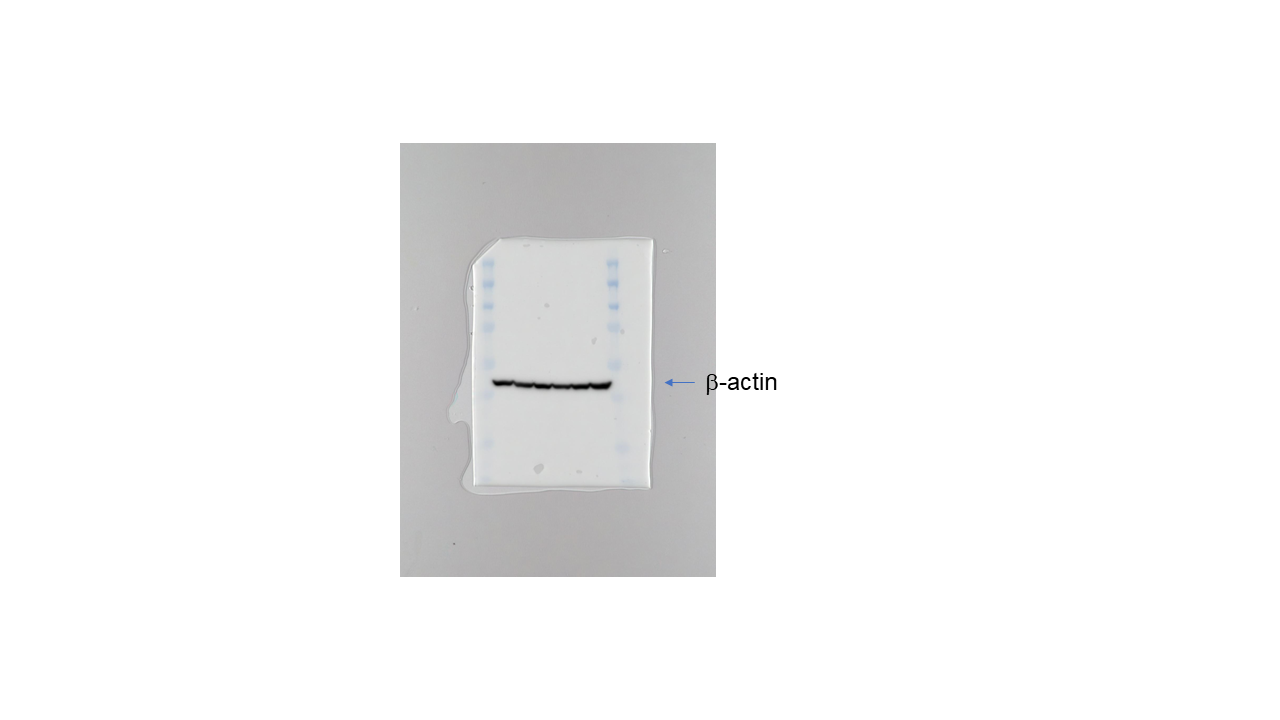

Supplement: Figure 5—figure supplement 1—source data 1. [file elife-83037-fig5-figsupp1-data1.zip › Figure 5-figure supplement 1-source data 1 2 indicated.TIF]

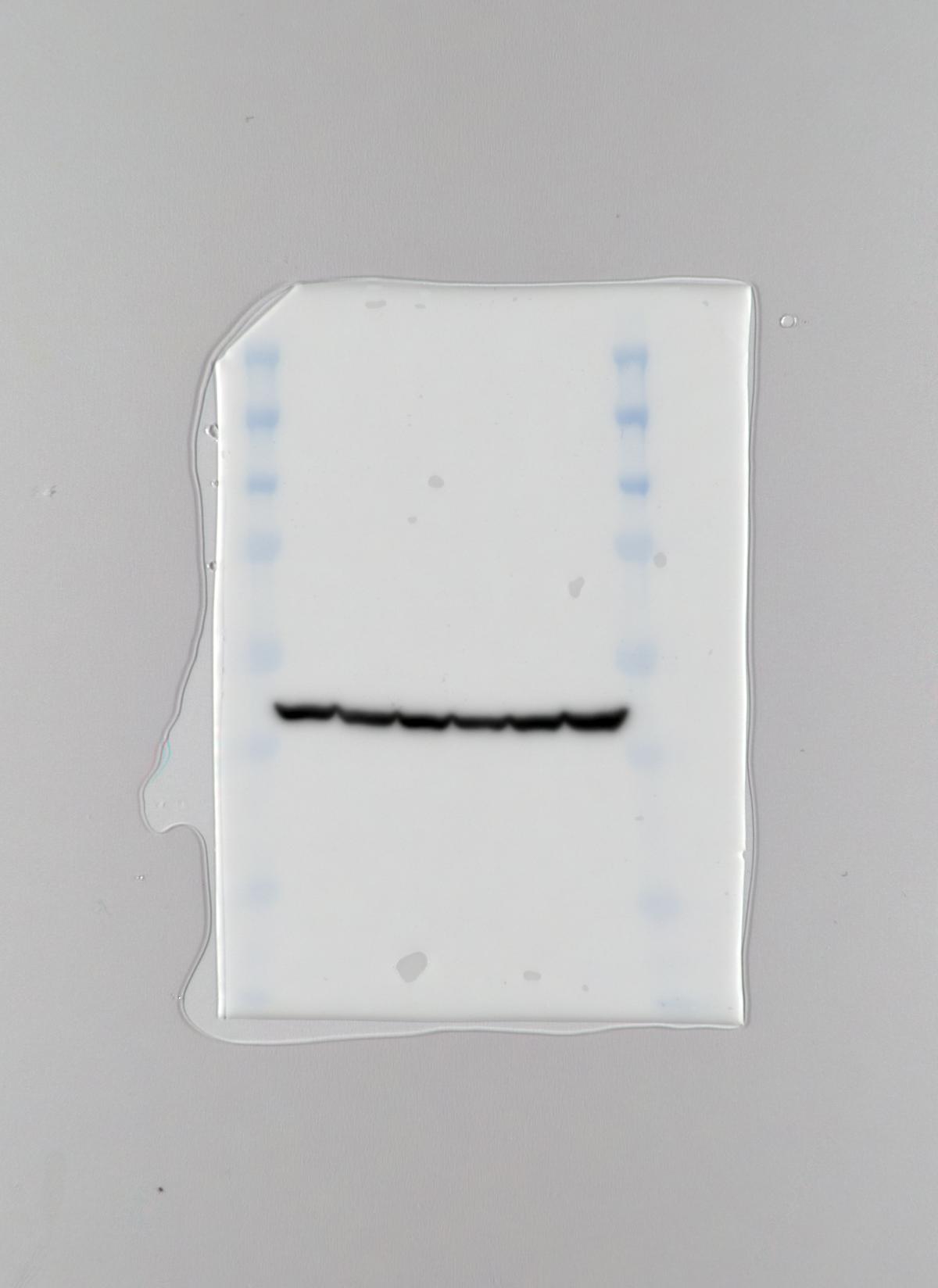

Supplement: Figure 5—figure supplement 1—source data 1. [file elife-83037-fig5-figsupp1-data1.zip › Figure 5-figure supplement 1-source data 1 2 raw.jpg]

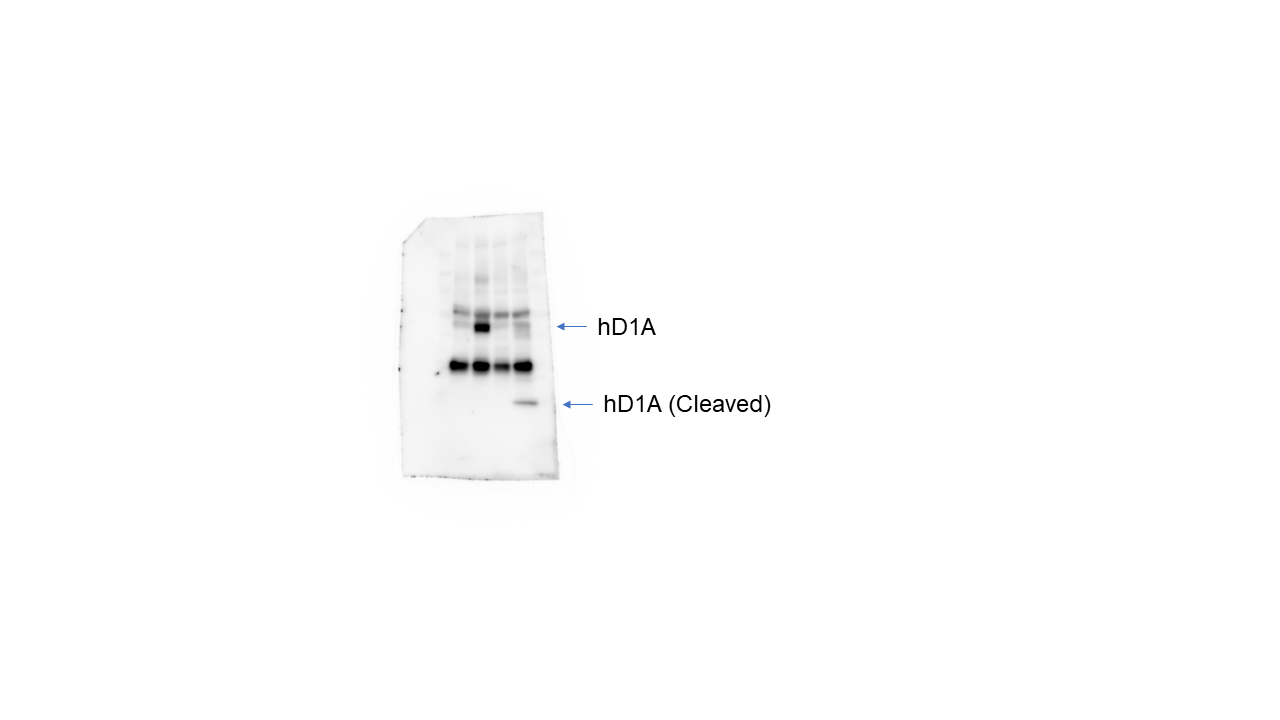

Supplement: Figure 6—source data 1. [file elife-83037-fig6-data1.zip › Figure 6-source data 1 indicated.tif]

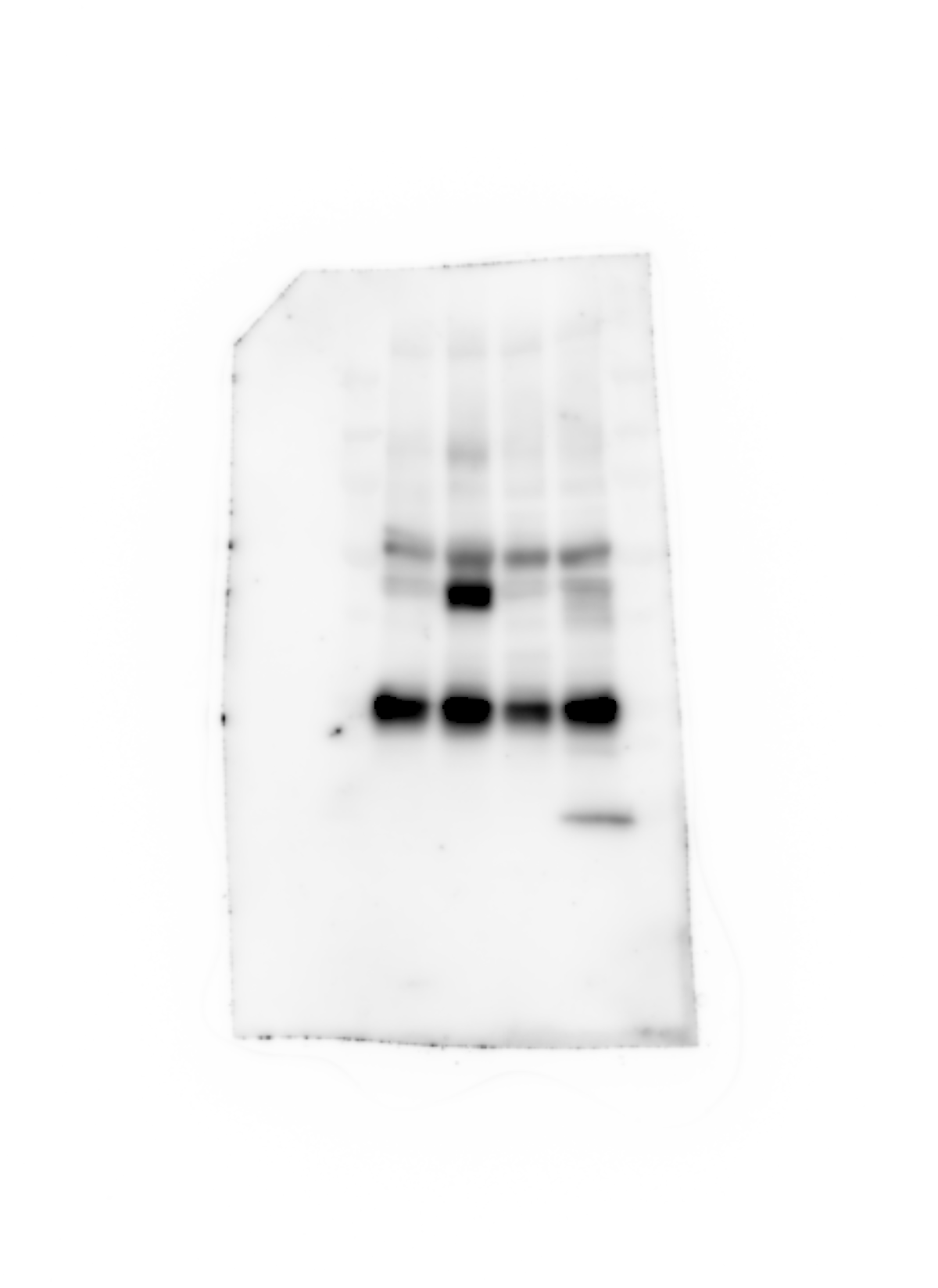

Supplement: Figure 6—source data 1. [file elife-83037-fig6-data1.zip › Figure 6-source data 1 raw.tif]
