# Supplementary material for: Human Dectin-1 is O-glycosylated and serves as a ligand for C-type lectin receptor CLEC-2
Source: eLife. 2022 Dec 8;11:e83037. doi: 10.7554/eLife.83037 (PMC9788829; doi:10.7554/eLife.83037)
Supplement: Supplementary file 1. [file elife-83037-supp1.docx]

**Supplementary file 1a**

**Table S1. List of primers and gRNAs used in this study.**

| Mice | Primers used for genotyping | |
| --- | --- | --- |
| Human *Dectin-1* transgenic | F | TGGTAATACTGGTGATAGCTGTGG |
|  | R | CTTTAGGAGATTAGAGCCCAGTTG |
| *Clec1b* KO | F | GCCATCATCTTTCTGACCTTGAGACCC |
|  | R | CCACGACAGATGTGCATTGCTTG |
|  | F | GCTCCCACAGCGGAACCTGC |
|  | R | CGCCTCGTGGCTGTTGCTTCC |
| *Podoplanin* KO | F | GCCTGTGGCTTCGAAGTTTTT |
|  | R | AACAAGTGCTGGTTGCCAATCAT |
| Cells | Primers used for cloning | |
| Mouse Dectin-1 | F | GGGAATTCATGAAATATCACTCTCATATAGAG |
|  | R | TTGCGGCCGCTTACAGTTCCTTCTCAC |
| Human Dectin-1 | F | TTTGAATTCATGGAATATCATCCTG |
|  | R | TGTTCTCGAGTTACATTGAAAACTTCTTCTCACAAATAC |
| Human Dectin-1A^ΔCRD^ | F | TTTGAATTCATGGAATATCATCCTG |
|  | R | TTTCTCGAGCCCTGTGGTTTTGACAGCTTTG |
| Human Dectin-1^T105A^ | F | GAAGACAGTGTGGCTCCTACCAAAGC |
|  | R | GCTTTGGTAGGAGCCACACTGTCTTC |
|  | F | TTTGAATTCATGGAATATCATCCTG |
|  | R | TGTTCTCGAGTTACATTGAAAACTTCTTCTCACAAATAC |
| Human Dectin-1^T107A^ | F | GAAGACAGTGTGACTCCTGCTAAAGCTGTCAAAACCACAG |
|  | R | CTGTGGTTTTGACAGCTTTAGCAGGAGTCACACTGTCTTC |
|  | F | TTTGAATTCATGGAATATCATCCTG |
|  | R | TGTTCTCGAGTTACATTGAAAACTTCTTCTCACAAATAC |
| Human Dectin-1.14A | F | TTTGAATTCATGGAATATCATCCTG |
|  | R | CCAAATAGCCATGGTACCCAGGACCACAG |
|  | F | GGGTTCTTTCCAGCCCTTGTCCTC |
|  | R | TGTTCTCGAGTTACATTGAAAACTTCTTCTCACAAATAC |
|  | F | GCTATTTGGAGAGCAAATGCCGGAGCGAACGCTTTGGAGAATGGCTACTTTCTAGCAAGAAATAAAGAGAACCACGCCCAACCCGCGCAAGCTGCTTTAGAAGACGCCGTGGCGCCTGCTAAAGCTGTCAAAGCAGCCGGG |
| Human Dectin-1.14A^105T^ | F | TGTGGTCCTGGGTACCATGGCTATTTGGAGAGCAAATGCCG |
|  | R | AGGACAAGGGCTGGAAAGAACCCCGGCTGCTTTGACAGCTTTAGCAGGAGTCACGGCGTCTTCTAAAG |
|  | F | TTTGAATTCATGGAATATCATCCTG |
|  | R | CCAAATAGCCATGGTACCCAGGACCACAG |
|  | F | GGGTTCTTTCCAGCCCTTGTCCTC |
|  | R | TGTTCTCGAGTTACATTGAAAACTTCTTCTCACAAATAC |
| Human Dectin-1^E101A/D102A^ | F | CTTTAGCAGCCAGTGTGACTCCTACC |
|  | R | GGTAGGAGTCACACTGGCTGCTAAAG |
|  | F | TTTGAATTCATGGAATATCATCCTG |
|  | R | TGTTCTCGAGTTACATTGAAAACTTCTTCTCACAAATAC |
| Human CLEC-2 | F | TTTGAATTCATGCAGGATGAAGATGGATACATCACC |
|  | R | TTTCTCGAGTTAAGGTAGTTGGTCCACCTTGG |
| Human CLEC-2/CD3ζ | F | TTTCTCGAGCAGCGCAATTACCTACAAGG |
|  | R | TTTCTCGAGAGGTAGTTGGTCCACCTTGG |
| Human CLEC-2^ΔCRD^/CD3ζ | F | TTTCTCGAGCAGCGCAATTACCTACAAGG |
|  | R | TTTCTCGAGTTTGAAAGTGCCCTTTAGTTCTGATTG |
| Human CLEC-2^R107A^/CD3ζ | F | CACAAACTGGGCATATTATGGAG |
|  | R | CTCCATAATATGCCCAGTTTGTG |
|  | F | TTTGAATTCATGCAGGATGAAGATGGATACATCACC |
|  | R | TTTCTCGAGTTAAGGTAGTTGGTCCACCTTGG |
| Human CLEC-2^R118A^/CD3ζ | F | GGGTTCTTCGCGCACAACTTAAC |
|  | R | GTTAAGTTGTGCGCGAAGAACCC |
|  | F | TTTGAATTCATGCAGGATGAAGATGGATACATCACC |
|  | R | TTTCTCGAGTTAAGGTAGTTGGTCCACCTTGG |
| Human CLEC-2^R152A^/CD3ζ | F | CATCAAAGCCGCGACTCATTTAATTCG |
|  | R | CGAATTAAATGAGTCGCGGCTTTGATG |
|  | F | TTTGAATTCATGCAGGATGAAGATGGATACATCACC |
|  | R | TTTCTCGAGTTAAGGTAGTTGGTCCACCTTGG |
| Human CLEC-2^R157A^/CD3ζ | F | TCATTTAATTGCTTGGGTCGGATTATC |
|  | R | GATAATCCGACCCAAGCAATTAAATGA |
|  | F | TTTGAATTCATGCAGGATGAAGATGGATACATCACC |
|  | R | TTTCTCGAGTTAAGGTAGTTGGTCCACCTTGG |
| Mouse CLEC-2/CD3ζ | F | TTTCTCGAGCAGCAAAAGTATCTACTGGCGG |
|  | R | TTTCTCGAGAAGCAGTTGGTCCACTC |
| Human Podoplanin | F | TTTCTCGAGATGTACCCATACGATGTTCCAGATTACGCTCCAGGTGCCGAAGATGATGTGGTGA |
|  | R | TTTGCGGCCGCTTAGGGCGAGTACCTTCCCGACATT |
| Human COSMC | F | TTTGAATTCATGCTTTCTGAAAGCAGC |
|  | R | TTTGCGGCCGCTCAGTCATTGTCAGAACC |
| Human ST6GalNAc4 | F | TTTGAATTCATGAAGGCTCCGGGTC |
|  | R | TTTCTCGAGCTACTCAGTCCTCCAGG |
| guide RNA |  |  |
| Clec1b-gRNA 1 |  | GGTTGTTGGACTCGTGGCTC |
| Clec1b-gRNA 2 |  | AAAGTATCTACTGGCGGAGA |
| Pdpn-gRNA 1 |  | GGTCCACATCTTGATCTCGT |
| Pdpn-gRNA 2 |  | TCTGGGTTTTGGGGAGCGTT |
| COSMC-gRNA |  | GAGTCTTTGGGCTGCAGTAA |
