# Supplementary material for: Human Dectin-1 is O-glycosylated and serves as a ligand for C-type lectin receptor CLEC-2
Source: eLife. 2022 Dec 8;11:e83037. doi: 10.7554/eLife.83037 (PMC9788829; doi:10.7554/eLife.83037)
Supplement: Supplementary file 2. [file elife-83037-supp2.docx]

**Supplementary file 1b**

**Table S2. List of antibodies used in this study.**

| Antibodies | SOURCE | IDENTIFIER |
| --- | --- | --- |
| FITC-conjugated mouse anti-human CD14 (HCD14) | Biolegend | Cat# 325603 |
| FITC-conjugated mouse anti-human TCRβ (IP26) | Biolegend | Cat# 306705 |
| PE-conjugated mouse anti-human CD19 (HIB19) | Biolegend | Cat# 302207 |
| Bv421-conjugated mouse anti-human CD41 (HIP8) | Biolegend | Cat# 303729 |
| FITC-conjugated mouse anti-human CLEC-2 (AYP1) | Biolegend | Cat# 372007 |
| APC-conjugated mouse anti-human Dectin-1 (259931) | R&D systems | Cat# FAB1859A |
| Mouse anti-human Dectin-1 (259931) | R&D systems | Cat# MAB1859 |
| Mouse anti-human Dectin-1A (BC2) | Dr Janet A. Willment. |  |
| Goat anti-human Dectin-1 polyclonal Ab (AF1859) | R&D systems | Cat# AF1859 |
| Rabbit anti-β-actin (13E5) | Cell Signaling TECHNOLOGY | Cat# 4970 |
| FITC-conjugated Hamster anti-mouse CD3e (145-2C11) | BD pharmingen | Cat# 553062 |
| Bv421-conjugated Rat anti-mouse CD19 (6D5) | Biolegend | Cat# 115537 |
| FITC-conjugated mouse anti-mouse CD45.1 (A20) | BD pharmingen | Cat# 553775 |
| APC-conjugated mouse anti-mouse CD45.2 (104) | BD pharmingen | Cat# 561875 |
| PE-conjugated rat anti-mouse CLEC-2 (17D9) | Biolegend | Cat# 146103 |
| Bv421-conjugated rat anti-mouse CD41 (MWReg30) | Biolegend | Cat# 133911 |
| APC-conjugated streptavidin | Invitrogen | Cat# 17-4317-82 |
| Mouse anti-HA (HA124) | Nacalai tesque | Cat# 06340-96 |
| APC-conjugated donkey anti rat IgG (H+L) | Jackson Immuno Research | Cat# 712-136-153 |
| HRP-Rabbit anti-goat IgG (H+L) | Invitrogen | Cat# 81-1620 |
| Mouse IgG_1_-kappa isotype control | BD pharmingen | Cat# 557273 |
| APC-conjugated mouse IgG_2a_-kappa isotype control | Biolegend | Cat# 400219 |
| Rat IgG_1_-kappa isotype control | BD pharmingen | Cat# 553921 |
| American hamster anti-mouse PECAM-1 (2H8) | Merck Millipore | Cat# MAB1398Z |
| Rabbit anti-mouse LYVE-1 | Relia Tech | Cat# 103-PA50AG |
| Rat anti-mouse TER-119 | BD Biosciences | Cat# 553670 |
| Rat anti-mouse CD41 | BD pharmingen | Cat# 553847 |
| Cy3-conjugated anti-Armenian hamster IgG | Jackson ImmunoResearch Laboratories | Cat# 127-165-160 |
| Cy5-conjugated anti-rat IgG | Jackson ImmunoResearch Laboratories | Cat# 112-175-143 |
| Alexa Fluor 488-conjugated anti-rabbit IgG | Invitrogen | Cat# A-11008 |
| Alexa Fluor 647-conjugated anti-rabbit IgG | Abcam | Cat# ab 150083 |
| Alexa Fluor 488-conjugated anti-Armenian hamster IgG | Abcam | Cat# ab 173003 |
| Alexa Fluor 555-conjugated anti-rat IgG | Invitrogen | Cat# A-21434 |
